# Variable Action Potential Backpropagation during Tonic Firing and Low-Threshold Spike Bursts in Thalamocortical But Not Thalamic Reticular Nucleus Neurons

**DOI:** 10.1523/JNEUROSCI.0015-17.2017

**Published:** 2017-05-24

**Authors:** William M. Connelly, Vincenzo Crunelli, Adam C. Errington

**Affiliations:** ^1^Neuroscience Division, School of Biosciences, Cardiff University, Cardiff CF10 3AX, United Kingdom,; ^2^Department of Physiology and Biochemistry, University of Malta, Msida MSD 2080, Malta,; ^3^Neuroscience and Mental Health Research Institute, School of Medicine, Cardiff University, Cardiff CF24 4HQ, United Kingdom, and; ^4^Eccles Institute of Neuroscience, The John Curtin School of Medical Research, Australian National University, Acton ACT 2601, Australia

**Keywords:** action potential, backpropagation, dendrite, low-threshold spike, thalamic reticular nucleus, thalamocortical

## Abstract

Backpropagating action potentials (bAPs) are indispensable in dendritic signaling. Conflicting Ca^2+^-imaging data and an absence of dendritic recording data means that the extent of backpropagation in thalamocortical (TC) and thalamic reticular nucleus (TRN) neurons remains unknown. Because TRN neurons signal electrically through dendrodendritic gap junctions and possibly via chemical dendritic GABAergic synapses, as well as classical axonal GABA release, this lack of knowledge is problematic. To address this issue, we made two-photon targeted patch-clamp recordings from rat TC and TRN neuron dendrites to measure bAPs directly. These recordings reveal that “tonic”' and low-threshold-spike (LTS) “burst” APs in both cell types are always recorded first at the soma before backpropagating into the dendrites while undergoing substantial distance-dependent dendritic amplitude attenuation. In TC neurons, bAP attenuation strength varies according to firing mode. During LTS bursts, somatic AP half-width increases progressively with increasing spike number, allowing late-burst spikes to propagate more efficiently into the dendritic tree compared with spikes occurring at burst onset. Tonic spikes have similar somatic half-widths to late burst spikes and undergo similar dendritic attenuation. In contrast, in TRN neurons, AP properties are unchanged between LTS bursts and tonic firing and, as a result, distance-dependent dendritic attenuation remains consistent across different firing modes. Therefore, unlike LTS-associated global electrical and calcium signals, the spatial influence of bAP signaling in TC and TRN neurons is more restricted, with potentially important behavioral-state-dependent consequences for synaptic integration and plasticity in thalamic neurons.

**SIGNIFICANCE STATEMENT** In most neurons, action potentials (APs) initiate in the axosomatic region and propagate into the dendritic tree to provide a retrograde signal that conveys information about the level of cellular output to the locations that receive most input: the dendrites. In thalamocortical and thalamic reticular nucleus neurons, the site of AP generation and the true extent of backpropagation remain unknown. Using patch-clamp recordings, this study measures dendritic propagation of APs directly in these neurons. In either cell type, high-frequency low-threshold spike burst or lower-frequency tonic APs undergo substantial voltage attenuation as they spread into the dendritic tree. Therefore, backpropagating spikes in these cells can only influence signaling in the proximal part of the dendritic tree.

## Introduction

Dendritic backpropagating action potentials (bAPs) have critical roles including induction of spike-timing-dependent synaptic plasticity, dendritic Ca^2+^ spike generation, and triggering dendritic neurotransmitter release (Larkum et al., 1999; Kampa et al., 2006, 2007; Acuna-Goycolea et al., 2008). In thalamocortical (TC) and thalamic reticular nucleus (TRN) neurons, despite imaging and computational investigations, the site of AP origin and the true extent of AP backpropagation throughout the dendritic tree remains unknown. In TC neurons, Ca^2+^-imaging studies (Crandall et al., 2010; Errington et al., 2010, 2012; Sieber et al., 2013) and a solitary dendritic recording study (limited for technical reasons to only ∼60 μm from the soma; Williams and Stuart, 2000) suggest that bAPs are strongly attenuated, with even spike trains unable to evoke significant distal dendritic Ca^2+^ influx. In TRN neurons, there is conflicting data, with some studies suggesting bAPs produce distance-dependent dendritic calcium transients (Δ[Ca^2+^]) similar to those in TC neurons (Cueni et al., 2008; Crandall et al., 2010) and others suggesting that bAPs can produce significant Δ[Ca^2+^] throughout the dendritic tree (Chausson et al., 2013). However, unlike TC neurons, no data from direct patch-clamp recordings on AP backpropagation in TRN dendrites are currently available. This is problematic because notable discrepancies have been reported previously between optical and electrical recording studies. For example, in cortical pyramidal neurons, very different estimates of AP backpropagation into basal dendrites were obtained using patch-clamp recordings (Nevian et al., 2007) and voltage-sensitive dye imaging (Antic, 2003). Although the underlying reasons are unclear, temporal undersampling might confound data from imaging experiments. Conversely, the main problem associated with electrical recording in thin dendrites, series resistance, can be adequately negated even for high-resistance recording electrodes (Nevian et al., 2007; Krueppel et al., 2011; Connelly et al., 2015). Furthermore, whereas Ca^2+^ imaging can report evoked Δ[Ca^2+^], these might not capture the full effects of bAP propagation because they also rely on dendritic Ca^2+^ channel distribution. For example, in the absence of distal dendritic Ca^2+^ channels, a bAP may not, on its own, induce Ca^2+^ entry but may still provide sufficient distal depolarization to relieve magnesium-dependent NMDA receptor block if appropriately timed relative to an EPSP. Therefore, the full impact of AP backpropagation in thalamic neurons can only be understood by investigating the voltage transients that APs produce throughout the dendritic tree.

From a physiological perspective, understanding the extent of backpropagation in TRN neurons is important because they signal, not only via classical axonal synapses, but also through dendritic electrical synapses mediated by Cx36-dependent gap junctions (Landisman et al., 2002) and, on the basis on structural evidence, GABAergic dendrodendritic synapses (Deschênes et al., 1985; Pinault et al., 1997). In other cell types, including interneurons of the dorsal LGN (Acuna-Goycolea et al., 2008), bAPs are effective in evoking dendritic neurotransmitter release; therefore, understanding backpropagation in TRN neurons will shed further light on the spatial extent of electrical and chemical signaling in TRN dendrites. Moreover, in both TC and TRN neurons, cortical and subcortical synaptic inputs have differential distribution patterns across the dendritic tree, with the former targeting more distal dendritic locations. Therefore, understanding the true extent of AP backpropagation in thalamic neurons is necessary to predict how bAPs might interact with specific synaptic inputs. Using two-photon targeted patch-clamp recordings from dendrites of TC and TRN neurons, we have for the first time measured AP backpropagation directly during both “tonic” and low-threshold-spike (LTS) “burst” firing modes. We find that APs are of axosomatic origin and undergo substantial voltage attenuation as they propagate into the dendrites of both cell types. Moreover, we find differences in bAP propagation within LTS bursts and between tonic and burst firing in TC neurons that are absent in TRN cells. Therefore, whereas TC and TRN neurons share some dendritic properties (Connelly et al., 2015), there are key differences in AP backpropagation between these cells.

## Materials and Methods

### 

#### 

##### Electrophysiology.

Coronal slices (300 μm) containing the dLGN and horizontal slices (250 μm) containing the TRN were prepared from postnatal day 20–25 (dLGN) and 17–21 (TRN) Wistar rats of either sex, deeply anesthetized using isoflurane, as described in Errington et al. (2010) with approval of the Cardiff University Research Ethics Committee and in accordance with the Home Office Animals (Scientific Procedures) Act 1986, United Kingdom. For recording, slices were transferred to a submersion chamber continuously perfused with warmed (33–4°C) aCSF containing the following (in mm): 125 NaCl, 2.5 KCl, 2 CaCl_2_, 1 MgCl_2_, 1.25 NaH_2_PO_4_, 25 NaHCO_3_, and 25 d-glucose with 305 mOsm at a flow rate of 2.5–3 ml/min. Somatic whole-cell patch-clamp recordings were made from TC and TRN neurons (visually identified by infrared gradient contrast video microscopy) using a Multiclamp 700B amplifier (Molecular Devices) and pipettes with resistances of 4–6 MΩ when filled with internal solution containing the following (in mm): 130 K-gluconate, 20 KCl, 10 HEPES, 0.16 EGTA, 2 Mg-ATP, 2 Na_2_-ATP, and 0.3 Na_2_-GTP at pH 7.3 and 295 mOsm and supplemented with 50 μm Alexa Fluor 594 (Invitrogen). Recording solutions did not routinely include any synaptic blocking drugs or other blocking toxins unless specifically indicated. Electrophysiological data were sampled at 20–50 kHz and filtered at 6 kHz. Somatic series resistance at the start of experiments was between 9 and 15 MΩ and varied ≤20% during recordings. Two-photon fluorescence microscopy using a Prairie Ultima (Prairie Technologies) microscope and titanium:sapphire pulsed laser (Chameleon Ultra II; Coherent Technologies) tuned to λ = 810 nm was combined with IR-scanning gradient contrast to make targeted dendritic patch-clamp recordings from thin (∼0.7–2 μm) dendrites of TC and TRN neurons (Connelly et al., 2015). Some data included in this study was obtained from neurons recorded previously in a separate study (Connelly et al., 2015). Dendritic recording pipettes were made from borosilicate glass capillaries (BF200-100-10; Sutter Instruments) and had resistances of 25–40 MΩ when filled with the internal recording solution described above. Although this resulted in dendritic recordings with high series resistance (40–110 MΩ), the ability of high-resistance recording electrodes to faithfully record dendritic membrane potentials has been demonstrated previously by others (Nevian et al., 2007; Bathellier et al., 2009; Larkum et al., 2009; Krueppel et al., 2011) and in our own laboratory (Connelly et al., 2015). As described previously (Connelly et al., 2015), somatic and dendritic bridge balance and pipette capacitance neutralization were carefully monitored and adjusted throughout experiments by application of low-frequency (50 Hz), low amplitude (10–40 pA) current steps. Although nonbursting TRN neurons have been identified previously (Contreras et al., 1992; Brunton and Charpak, 1997; Lee et al., 2007), for the purposes of this study, all TRN cells recorded from were capable of producing LTS bursts in response to both depolarizing and hyperpolarizing current injections.

##### Measurement of AP properties.

**Figure 1.**
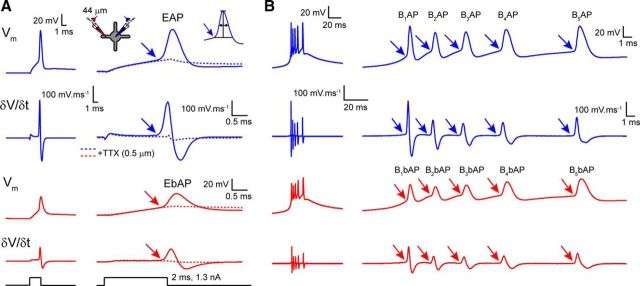
Measuring tonic and burst AP properties using the first temporal derivative of the voltage signal. ***A***, Membrane potential (*V*_m_) and first temporal derivative (δ*V/*δ*t*) of an averaged somatically evoked AP simultaneously recorded in the soma (EAP, blue) and dendrite (EbAP, red) of a typical TC neuron. The expanded view illustrates a clear inflection point in both *V*_m_ and δ*V/*δ*t* in the soma and dendrites (indicated by arrows) that indicate AP onset. Dashed lines show *V*_m_ and δ*V/*δ*t* produced by the same current injection step in the presence of TTX, highlighting the onset of the active regenerative Na^+^ spike compared with the passive current injection response. The inset illustrates the method used to measure spike half-width, which was defined as the spike width at half-maximal amplitude where amplitude is the difference between δ*V/*δ*t* threshold and spike peak. ***B***, *V*_m_ and first temporal derivative (δ*V/*δ*t*) of a typical LTS burst recorded in the soma (B_X_AP, blue) and dendrite (B_X_bAP, red) of the same TC neuron as in ***A***. Individual AP onset can be differentiated clearly from the underlying slow low threshold Ca^2+^ potential in both the soma and dendrites using the marked change in δ*V/*δ*t* present with each spike (indicated by arrows).

Tonic APs and LTS burst-associated APs (LTS-burst) were evoked using three different stimulation protocols and simultaneously recorded in the soma (depicted by blue traces in figures) and dendrites (depicted by red traces in figures) of TC and TRN neurons. First, to produce single tonic APs, TC and TRN neurons were depolarized to approximately −55 mV using direct current injection to inactivate the majority of T-type Ca^2+^ channels and to prevent triggering LTS bursts and brief current injection steps of between 1 and 2 nA and 2 ms duration were injected via the somatic recording electrode (Fig. 1*A*). Hereafter, when recorded at the soma, these will be referred to as evoked APs (EAPs) and when recorded in the dendrites as evoked bAPs (EbAPs). The average of a minimum of 10 EAPs/EbAPs were used to measure AP properties at each different dendritic location. To investigate frequency-dependent effects on AP backpropagation, EAPs were also evoked as trains of 5 spikes at 10, 30, and 50 Hz. Second, trains of APs typical of the prolonged tonic firing observed in TC and TRN neurons in behaving animals were analyzed. These tonic APs will hereafter be referred to as TAPs when recorded at the soma and TbAPs when recorded in the dendrites. TAPs were evoked from the resting membrane potential of TC and TRN neurons by injecting long (0.5–1 s) depolarizing current steps (200 pA) via the somatic recording electrode. Spikes that were not associated with the initial LTS burst were considered TAPs (dashed boxes in Figs. 4*C*, 5*C*) and included in the analysis. TAPs and TbAPs were detected using a threshold-crossing detection method and aligned to threshold before averaging. A minimum of 10 TAPs/TbAPs were averaged for measurement of spike properties at each different recording location. Finally, backpropagation of LTS-burst-associated APs was assessed for spike bursts evoked by 1-s-long hyperpolarizing current injections from resting membrane potential in TC neurons and from −55 mV in TRN neurons. Hereafter, these LTS-burst-associated APs are referred to as B_X_APs for somatic recordings and B_X_bAPs for dendritic recordings, where the subscript letter “X” refers to the temporal position of the individual AP within the LTS burst (i.e., B_1_AP/B_1_bAP is the first spike in an LTS burst). To overcome problems associated with measuring the properties of individual spikes within the LTS burst, we applied a method using the first temporal derivative of the recorded voltage signals. For all spikes, including EAPs/EbAPs, TAPs/TbAPs, and B_X_APs/B_X_bAPs, the first temporal derivative (δ*V/*δ*t*) of the somatic and dendritic spikes were calculated using PClamp 10 (Molecular Devices) to determine spike threshold (Fig. 1). Threshold was determined through observation of a clear inflection in δ*V/*δ*t* signifying spike onset (typically between 5 and 15 mV/ms; Fig. 1*A*) and this was classified as the baseline. Spike amplitude was then determined as the difference between the baseline value and the peak voltage of the spike. AP half-width was determined by measuring the spike width at the value 50% between the baseline and peak spike voltage. Spike latency was measured as the difference between the peak of the somatic and dendritic APs. Using this approach, it was possible to measure the properties of individual spikes during LTS bursts because the rate of membrane potential change during an AP is significantly greater than that of the underlying low-threshold Ca^2+^ spike (Fig. 1*B*). To quantify the relationship between measured AP amplitudes with respect to the distance between the somatic and dendritic recording location, data were fit with a mono-exponential function of the form *f*(*x*) = exp(−*x*/λ_eff_), to yield the “effective space constant” λ_eff_. It is important to note, as described by others (Bathellier et al., 2009), that this differs from the canonical electrotonic length constant that appears in the cable equation.

To test for the presence of local dendritic Na^+^ spikes, dendritic EPSPs were evoked by injecting EPSC-like currents of increasing amplitude through the dendritic recording electrode. Injected EPSCs were designed to produce EPSPs similar to those recorded in previous studies (Connelly et al., 2016). We tested two injected EPSCs, one with a fast (2 ms) decay time constant and one with a slower (5 ms) decay time constant, because longer EPSPs have been shown previously to more readily evoke dendritic Na^+^ spikes in cortical pyramidal neurons (Bathellier et al., 2009). To compare the size of measured dendritic EPSPs recorded in response to EPSC-like current injection versus the size of the expected EPSPs generated by EPSCs with linearly increasing amplitude, expected EPSPs were calculated by multiplying the voltage response of the smallest EPSP (EPSP_1_) evoked by the minimal EPSC (EPSC_1_ = 10 pA) by the necessary scaling factor (i.e., measured EPSP_1_ was multiplied by three to estimate the expected EPSP evoked by a 30 pA EPSC).

##### Data analysis and statistics.

Throughout this study, a previously used schematic illustration scheme (Connelly et al., 2015) is used to distinguish experiments performed in TC and TRN neurons. For clarity, these schematic symbols are redefined in Figures 4 (TC) and 5 (TRN). Distances between the dendritic and somatic recording electrode were measured *post hoc* from 2D maximum intensity projections of 3D *Z*-series image stacks (120–150 images at 1 μm step size) collected at the end of each experiment as described in Errington et al. (2010). The dendrites recorded from were constrained to a narrow optical plane (≤20 μm *Z* variance) parallel to the surface of the slice. Data analysis was performed using pClamp 10 (Molecular Devices), Excel (Microsoft), ImageJ, and Prism (GraphPad) software. Statistical testing was by unpaired *t* test or repeated-measures ANOVA where appropriate and all data values are presented as mean ± SEM. Quoted values for *n* are the number of neurons in each group.

## Results

### AP and dendritic sodium spike initiation in TC and TRN neurons

Both TC and TRN neurons characteristically fire APs in two distinct patterns, namely tonic and burst mode, depending upon their membrane potential, which *in vivo* is dependent on behavioral state (Sherman, 2001). In burst mode, which most typically, but not exclusively, occurs during low vigilance states and sleep, APs in both TC and TRN neurons are fired on the crest of a Ca^2+^ LTS at frequencies of several hundred hertz with interspike intervals as short as 2 ms. In contrast, during periods of wakefulness, APs are fired in tonic mode at frequencies typically <50 Hz. This behavioral-state-dependent change in firing pattern is thought to primarily allow LTS burst spikes to transmit different signals to postsynaptic cortical targets compared with tonic spikes (Swadlow and Gusev, 2001). Nonetheless, these firing modes might also facilitate, via AP backpropagation, transmission of variable information about thalamic neuronal output into the dendritic tree and selective activation of specific dendritic signaling mechanisms. Therefore, we performed two-photon targeted paired somatodendritic recordings (Connelly et al., 2015) to assess AP backpropagation definitively in TC and TRN neurons during tonic and burst firing.

**Figure 2.**
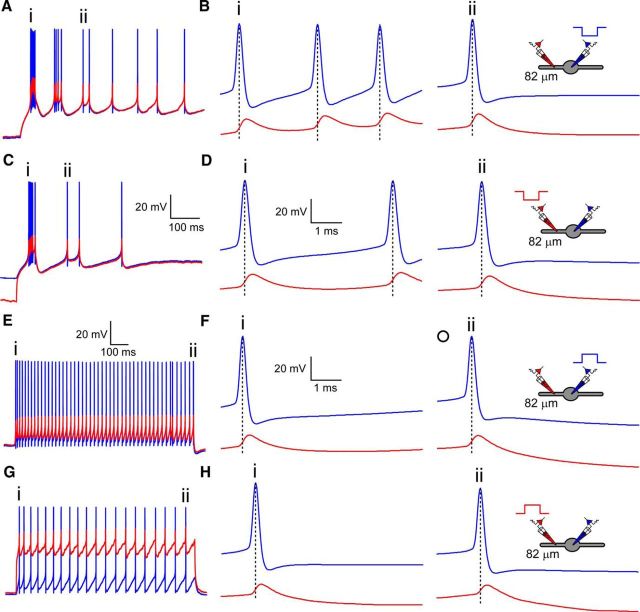
Axosomatic initiation of tonic and burst APs in thalamic reticular nucleus neurons. ***A***, Simultaneous somatic (blue) and dendritic (red, 82 μm) recording of rhythmic LTS AP bursts evoked by hyperpolarizing somatic current injection from resting membrane potential (∼−70 mV). Scale as in ***C***. ***B***, Somatic and dendritic APs occurring during the first (***i***) and third (***ii***) LTS AP bursts. Dashed lines illustrate the delay between the peak of the somatic and dendritic spike. Scale as in ***D***. ***C***, As in ***A***, but for rebound LTS AP bursts evoked by hyperpolarizing current injection into the dendrites. Dendritic current injection produces significant dendritic hyperpolarization. ***D***, As in ***B***, but for dendritic current injection. ***E***, Simultaneous somatic (blue) and dendritic (red) recording from the same neuron as in ***A*** held at ∼−55 mV using d.c. injection. Tonic APs were evoked by somatic depolarizing current injections steps. ***F***, Somatic and dendritic APs occurring at the start (***i***) and end (***ii***) of a tonic AP train. Dashed lines illustrate the delay between the peak of the somatic and dendritic spike. ***G***, As in ***E***, but for tonic APs evoked by dendritic depolarizing current injection. Dendritic current injection produces significant dendritic depolarization. Note that dendritic amplitude of APs evoked by dendritic current injection does not differ from dendritic APs evoked by somatic current injection. Scale as in ***E***. ***H***, As in ***F***, but for dendritic current injection. Scale as in ***F***.

First, the ability to record the dendritic membrane potential directly afforded us the opportunity to investigate experimentally the subcellular AP initiation site in TC and TRN neurons. Consistent with the only previous study to investigate AP backpropagation directly (Williams and Stuart, 2000), our data confirm that, in TC neurons, APs originate in the axosomatic region before backpropagating into dendrites with a tangible delay regardless of whether APs are fired in the tonic mode (*n* = 25) or are associated with LTS bursts (*n* = 36). In every TC neuron from which we recorded, APs were always recorded first at the somatic recording electrode, followed by the dendritic recording electrode (data not shown). However, for TRN neurons, no equivalent direct measure of the AP initiation site exists. Therefore, we examined AP initiation during rebound LTS bursts by injecting hyperpolarizing current via the somatic or dendritic recording electrode. APs associated with the first rebound LTS, evoked by either somatic (Fig. 2*A*,*B*) or dendritic (Fig. 2*C*,*D*) hyperpolarization, were always recorded first at the somatic electrode and subsequently the dendritic electrode (*n* = 16). In the majority of TRN neurons, after strong membrane potential hyperpolarization, rhythmic sequences of LTS bursts occur (Fig. 2*A*,*C*). It has been suggested that these secondary LTS bursts differ from the primary burst in respect of the relative contribution of R-type (Ca_V_2.3) versus T-type Ca^2+^ (Ca_V_3.2 and 3.3) channels (Zaman et al., 2011). However, our recordings show that APs associated with secondary LTS bursts follow the same pattern as observed for the initial LTS burst, being observed first in the soma and then dendrite with the same latency between AP peaks for both bursts (Fig. 2*B*,*D*). Similarly to LTS burst-associated APs, tonic APs were also always recorded first at the somatic recording electrode, followed by the dendritic electrode (Fig. 2*E*,*F*), even when evoked by injecting depolarizing current into the dendrites (Fig. 2*G*,*H*). Therefore, by recording directly from dendrites, these data demonstrate for the first time that APs in TRN neurons are initiated in the axosomatic region and not in dendrites.

**Figure 3.**
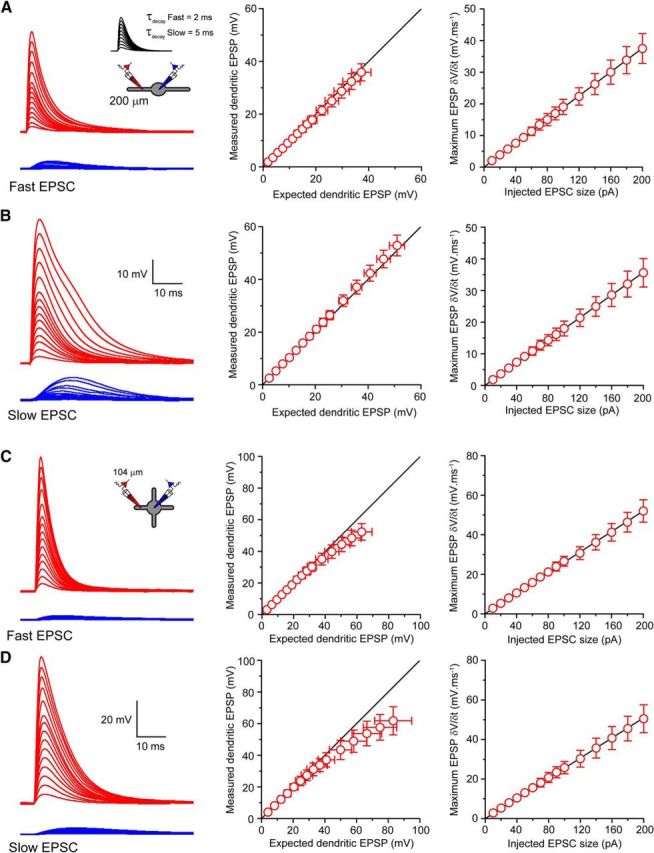
Absence of local dendritic sodium spike initiation in thalamocortical and thalamic reticular nucleus neurons. ***A***, Voltage at the soma (blue) and dendrite (red) resulting from fast EPSC-like current injections of increasing size into a distal dendrite (200 μm) of a TRN neuron. Even large (>40 mV) dendritic depolarizations cannot produce local dendritic Na^+^ spikes. Plots demonstrate the linear relationship between the measured dendritic EPSP and the expected EPSP and between the maximum δ*V/*δ*t* of dendritic EPSPs and the size of the injected dendritic EPSC. ***B***, As in ***A***, but for slow dendritic EPSC injections. Slow EPSCs induce larger dendritic EPSPs but still reveal no evidence for the presence of dendritic Na^+^ spikes. ***C***, Voltage at the soma (blue) and dendrite (red) resulting from fast EPSC-like current injections of increasing size into a distal dendrite (104 μm) of a TC neuron. Plots demonstrating the weakly sublinear relationship between measured and expected dendritic EPSPs in TC neurons and the linear increase in maximum dendritic EPSP δ*V/*δ*t* with increasing EPSC size. ***D***, As in ***C***, but for slow dendritic EPSC injections.

Nonetheless, in thin dendrites of other neurons, notably cortical and hippocampal pyramidal neurons (Golding and Spruston, 1998, Bathellier et al., 2009), voltage-dependent dendritic Na^+^-spikes can be initiated locally. To test whether TC and/or TRN dendrites support dendritic Na^+^ spike initiation, we injected EPSC-like currents into distal dendrites (TC: 100–156 μm, *n* = 9, TRN: 129–200 μm, *n* = 3; Bathellier et al., 2009; Ledergerber and Larkum 2010) and compared the recorded dendritic EPSPs with the expected EPSP, estimated using the amplitude of the smallest recorded subthreshold EPSP (see Materials and Methods). In TRN neurons, fast dendritically injected EPSCs produced local EPSPs with measured amplitudes that were linearly related to the expected EPSP (*n* = 3; Fig. 3*A*). Moreover, we observed no indication of a nonlinear step in EPSP amplitude or maximum δ*V/*δ*t* (Fig. 3*A*) indicative of Na^+^ spikes as described in dendrites of other neurons (Bathellier et al., 2009; Ledergerber and Larkum 2010). Injection of slower dendritic EPSCs, although producing larger local EPSPs, was also incapable of evoking nonlinear responses (Fig. 3*B*). Despite the small number of observations for EPSP-like current injections into the distal dendrites of TRN neurons, the highly linear relationship between the measured local EPSP and the expected EPSP support the conclusion that Na^+^ spikes are not generated in TRN dendrites. This is supported by the fact that we did not observe the occurrence of spikes in dendrites before the soma even with larger and longer depolarizing steps (Fig. 2*G*). In TC neurons (*n* = 9), we observed a weakly sublinear relationship between measured and expected dendritic EPSP amplitude for both fast (Fig. 3*C*) and slow (Fig. 3*D*) injected EPSCs. Finally, for TC neurons, maximum EPSP δ*V/*δ*t* was linearly related to the size of the injected EPSC (Fig. 3*C*,*D*). Therefore, distal dendrites of both TC and TRN neurons are incapable of initiating local dendritic voltage-dependent Na^+^-spikes.

### Backpropagation of tonic APs in TC and TRN neuron dendrites

**Figure 4.**
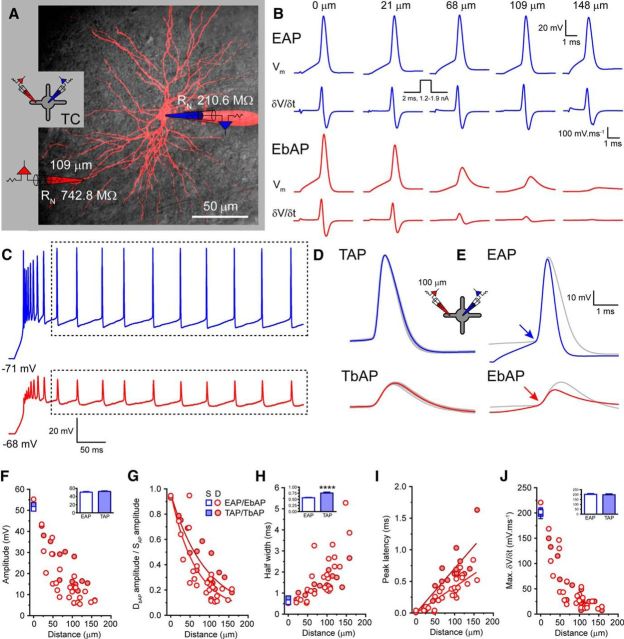
Tonic AP backpropagation in TC neurons. ***A***, Overlay of two-photon fluorescence maximum intensity projection and scanning gradient contrast image of a dLGN TC neuron showing the location of the somatic (blue) and dendritic (red) recording pipettes. Inset, Schematic of the recording configuration illustrating the neuron recorded from (TC) and the placement of electrodes. This schematic is used throughout to indicate that the illustrated data are drawn from TC neuron recordings. ***B***, *V*_m_ and δ*V/*δ*t* for APs evoked by brief somatic current injections recorded in the dendrites (EbAP, red) of five different TC neurons at increasing distances from the soma (EAP, blue). ***C***, Typical train of tonic APs recorded in the soma (TAP, blue) and dendrites (TbAP, red) of a TC neuron evoked by a prolonged somatic current injection step. Dashed boxes enclose those spikes included for analysis. ***D***, Gray traces show overlaid the individual TAPs and TbAPs measured from the period indicated by the dashed box in ***C***. The blue and red traces are the average TAP and TbAP, respectively. ***E***, EAP (blue) and EbAP (red) recorded at the same dendritic location in the same TC neuron compared with the average TAP and TbAP (gray traces).(***F***) Amplitude of EAPs (open blue squares) and EbAPs (open red circles) and TAPs (filled blue squares) and TbAPs (filled red circles) versus recording distance from soma. For clarity, somatic values are mean ± SEM. Inset bar chart shows no difference in mean somatic EAPs and TAP amplitude. ***G***, Normalized EbAP (EbAP/EAP) and TbAP (TbAP/TAP) amplitude versus recording distance from soma. ***H***, EAP and EbAP and TAP and TbAP half-width versus recording distance from soma. Inset shows the significant increase in somatic half-width for TAPs versus EAPs. ***I***, Peak latency between EAPs and EbAPs and TAPs and TbAPs versus distance from the soma. ***J***, Maximum δ*V/*δ*t* versus distance from the soma for EAPs and EbAPs and TAPs and TbAPs. Inset shows no significant difference in the somatic maximum δ*V/*δ*t* for TAPs versus EAPs.

**Figure 5.**
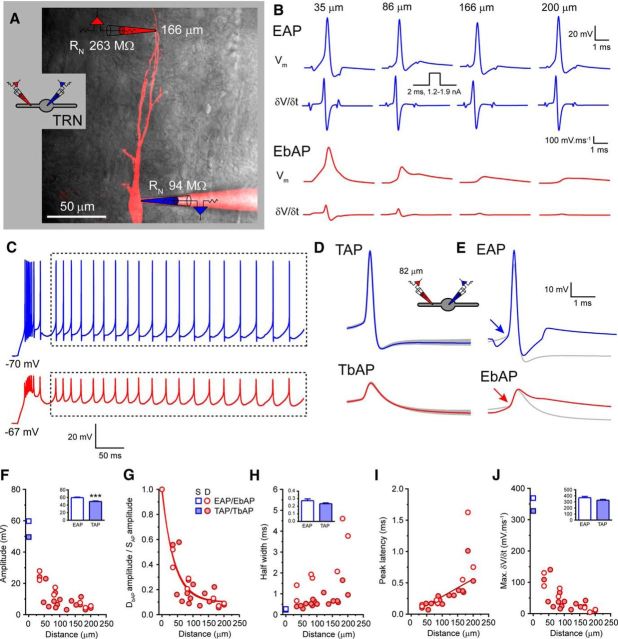
Tonic AP backpropagation in TRN neurons. ***A***, Overlay of two-photon fluorescence maximum intensity projection and scanning gradient contrast image of a somatosensory TRN neuron showing the location of the somatic (blue) and dendritic (red) recording pipettes. Inset, Schematic of the recording configuration illustrating the neuron recorded from (TRN) and the placement of electrodes. This schematic is used throughout to indicate that the illustrated data are drawn from TRN neuron recordings. ***B***, *V*_m_ and δ*V/*δ*t* for APs evoked by brief somatic current injections recorded in the dendrites (EbAP, red) of four different TRN neurons at increasing distances from the soma (EAP, blue). ***C***, Typical train of tonic APs recorded in the soma (TAP, blue) and dendrites (TbAP, red) of a TRN neuron evoked by a prolonged somatic current injection step. Dashed boxes enclose those spikes included for analysis. ***D***, Gray traces show overlaid the individual TAPs and TbAPs measured from the period indicated by the dashed box in ***C***. The blue and red traces are the average TAP and TbAP, respectively. ***E***, EAP (blue) and EbAP (red) recorded at the same dendritic location in the same TRN neuron compared with the average TAP and TbAP (gray traces). ***F***, Amplitude of EAPs (open blue squares) and EbAPs (open red circles) and TAPs (filled blue squares) and TbAPs (filled red circles) versus recording distance from soma. For clarity, somatic values are mean ± SEM. Inset bar chart shows significant difference in mean somatic EAPs and TAP amplitude. ***G***, Normalized dendritic EbAP (EbAP/EAP) and TbAP (TbAP/TAP) amplitude versus recording distance from soma. ***H***, EAP and EbAP and TAP and TbAP half-width versus recording distance from soma. Inset shows no significant increase in somatic half-width for TAPs versus EAPs. ***I***, Peak latency between EAPs and EbAPs and TAPs and TbAPs versus distance from the soma (***J***) Maximum δ*V/*δ*t* versus distance from the soma for EAPs and EbAPs and TAPs and TbAPs. Inset shows no significant difference the in somatic maximum δ*V/*δ*t* for TAPs versus EAPs.

Having established their axosomatic origin, we performed a detailed examination of AP backpropagation in TC and TRN neuron dendrites. First, we evoked APs in the tonic mode, which is typified by a depolarized resting membrane potential and commonly associated *in vivo* with wakefulness. To do this, we recorded membrane potential simultaneously at the soma and dendrites (Figs. 4*A*, 5*A*) while evoking spikes using two distinct current injection protocols. First, to measure backpropagation of EAPs, neurons were depolarized to −55 mV using d.c. injection and brief somatic current injections (2 ms, 1–2 nA) were used to trigger spikes (Figs. 4*B*, 5*B*). The mean amplitude of EAPs was 50.7 ± 1.5 mV for TC neurons (*n* = 25; Fig. 4*B*,*F*) and 59.8 ± 1.3 mV for TRN neurons (*n* = 7; *p* < 0.01, unpaired *t* test; Fig. 5*B*,*F*). Consistent with differences observed between other excitatory and inhibitory neurons, EAPs of GABAergic TRN neurons were markedly faster than those of TC neurons having significantly shorter half-widths (TRN: 0.27 ± 0.02 ms, *n* = 7; TC: 0.57 ± 0.01 ms, *n* = 25; *p* < 0.0001 unpaired *t* test; Figs. 4*B*,*E*,*H*, 5*B*,*E*,*H*) and greater maximum δ*V/*δ*t* (TRN: 369.0 ± 21.3 mV · ms^−1^, *n* = 7; TC: 202.0 ± 8.4 mV · ms^−1^, *n* = 25; *p* < 0.0001, unpaired *t* test; Figs. 4*B*,*E*,*J*, 5*B*,*E*,*J*). In both TC and TRN neurons, EAPs showed considerable distance-dependent voltage attenuation as they backpropagated into dendrites (Figs. 4*B*,*E*,*F*, 5*B*,*E*,*F*). To quantify the spatial extent of AP backpropagation, we fitted plots of EbAP amplitude, normalized to EAP amplitude, against distance of the dendritic recording location from the soma with single exponential functions. These fits revealed λ_eff_ for EAP attenuation of 64 μm for TC neurons (Fig. 4*G*) and 37 μm for TRN neurons (Fig. 5*G*). Therefore, for EAPs, attenuation of spike amplitude is greater per micrometer in TRN neurons than in TC neurons and, for both cells, bAP attenuation is steeper than that found in other neurons, for example, cortical pyramidal cells (Nevian et al., 2007). Consistent with AP backpropagation in other neurons (Nevian et al., 2007, Larkum et al., 2009, Bathellier et al., 2009; Ledergerber and Larkum 2010), as spikes propagated into the dendritic tree of both TC and TRN neurons, they became markedly broader and slower, as shown by distance-dependent increases in AP half-width and reduction in maximum δ*V/*δ*t* (Figs. 4*A*,*H*,*J*, 5*A*,*H*,*J*). Finally, as described above, in TC and TRN neurons, the EAP peak was always observed before the peak of the EbAP. In TC neurons, a linear regression fit to the peak spike latency versus distance from the soma revealed an average conduction velocity for EbAPs of 243.1 μm/ms (*n* = 25; Fig. 4*I*). For TRN neurons, due to the relatively small number of recordings and strong bAP attenuation, it was difficult to estimate conduction velocity of EbAPs accurately. Nonetheless, by excluding a single outlying data point from our analysis (Fig. 5*I*), we were able to estimate a conduction velocity of 295.7 μm/ms (*n* = 6) for EbAPs in TRN neurons (Fig. 5*I*). This measurement is similar to that for recorded for TbAPs (described below) in TRN dendrites, for which we have a larger dataset, and demonstrates that bAPs propagate more rapidly in TRN versus TC dendrites.

Because, *in vivo*, both TC and TRN neurons often respond to stimuli by firing trains of TAPs, we next investigated spike backpropagation during such tonic firing by injecting prolonged somatic depolarizing current steps. APs associated with the initial LTS burst were excluded from the analysis (Figs. 4*C*, 5*C*). In both TC and TRN neurons, we found no apparent activity-dependent changes in individual spike properties of TAPs or TbAPs throughout the spike train. Figures 4*D* and 5*D* show the average TAP (blue) and TbAP (red) overlaid onto the individual APs (gray) occurring during the trains shown for TC (Fig. 4*C*) and TRN (Fig. 5*C*) neurons, respectively. Interestingly, in TC neurons, we found notable differences between TAPs and EAPs. Both the amplitudes (52.6 ± 1.2 mV, *n* = 18, *p* > 0.05, unpaired *t* test; Fig. 4*E*,*F*) and maximum δ*V/*δ*t* (198.0 ± 9.1 mV · ms^−1^, *n* = 18, *p* < 0.05, unpaired *t* test; Fig. 4*E*,*J*) of TAPs were not significantly different from those of EAPs. However, the half-widths of TAPs (0.76 ± 0.04 ms, *n* = 18) were significantly greater than those of EAPs (0.57 ± 0.01 ms, *n* = 25, *p* < 0.001, unpaired *t* test; Fig. 4*D*,*E*,*H*). Coincident with this, TbAPs, on average, had significantly larger amplitudes than EbAPs (TbAP: 23.1 ± 3.0 mV, *n* = 25, EbAP: 15.7 ± 2.1 mV, *n* = 18, *p* < 0.05, unpaired *t* test; Fig. 4*E*,*F*), having an λ_eff_ of 91 μm (Fig. 4*G*). Moreover, the peak latency between TAPs and TbAPs in TC neurons was greater than the latency between EAPs and EbAPs, with an average conduction velocity for TbAPs of 141.9 μm/ms (Fig. 4*I*). Therefore, it appears that slower TAPs, backpropagate more efficiently into the dendrites of TC neurons than EAPs, in which somatic half-widths are shorter. The reason for this difference in TAP and EAP half-width is due to the membrane potential from which they are evoked. On average, the resting membrane potential before the initiation of EAPs was notably more hyperpolarized (∼−55 mV; Fig. 4*B*,*E*) and further from spike threshold (∼−40 mV) than for TAPs (∼−45 to −40 mV; Fig. 4*C*,*D*). Therefore, it appears that, in TC neurons, AP shape and duration is effected by resting membrane potential before spike onset and that this, in turn, determines the efficiency of spike backpropagation into dendrites.

In comparison, in TRN neurons, we did not see differences between EAPs and TAPs. Although TAPs in TRN neurons had significantly smaller amplitudes than EAPs (TAP: 49.6 ± 1.4 mV, *n* = 16, *p* < 0.001, unpaired *t* test; Fig. 5*E*,*F*), their half-widths were not significantly different (TAP: 0.23 ± 0.01 ms, *n* = 16, EAP: 0.27 ± 0.02 ms, *n* = 7, *p* > 0.05, unpaired *t* test; Fig. 5*D*,*E*,*H*) despite being evoked from different membrane potentials (TAP ∼−45 to −40 mV, Fig. 5*D*, EAP ∼−60 to −55 mV, Fig. 5*E*). Therefore, in TRN cells, the mean amplitudes of TbAPs were not significantly different from those of EbAPs (TbAP: 9.2 ± 1.6 mV, *n* = 16, EbAP: 14.0 ± 3.4 mV, *n* = 7, *p* < 0.05, unpaired *t* test; Fig. 5*D–F*), with TbAPs having an λ_eff_ of 40 μm, almost identical to that of EbAPs (Fig. 5*G*). The conduction velocity of TbAPs in TRN dendrites was 424 μm/ms (*n* = 16; Fig. 5*I*). Therefore, in TRN neurons, the preceding membrane potential has little effect on the shape and duration of individual APs and, as a result, their backpropagation into the dendritic tree.

**Figure 6.**
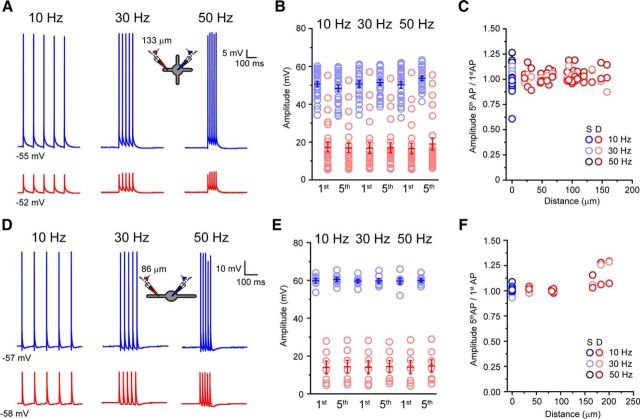
Absence of frequency-dependent attenuation of dendritic APs in TC and TRN neurons. ***A***, Typical trains of five evoked APs at 10, 30, and 50 Hz recorded at the soma (blue) and dendrite (red) of a TC neuron. ***B***, Plot showing the amplitude of the first and fifth APs in a train of five recorded at the soma and dendrites at 10, 30, and 50 Hz. Light blue circles are individual EAPs, light red circles are individual EbAPs, and blue and red bars are mean ± SEM. ***C***, Ratio of the amplitude of the fifth AP versus the first AP recorded at the soma (fifth EAP/first EAP) or dendrites (fifth EbAP/first EbAP) plotted against recording distance from the soma for each spike train frequency. Inset indicates the recording location and frequency of evoked spikes. ***D***, As in ***A***, but for a TRN neuron. ***E***, As in ***B***, but for a TRN neuron. ***F***, As in ***C***, but for a TRN neuron.

Finally, we examined two physiological mechanisms previously associated with bAPs, namely frequency-dependent bAP attenuation (Jung et al., 1997) and frequency-dependent Ca^2+^ electrogenesis (Larkum et al., 1999; Ledergerber and Larkum, 2010). To do this, we evoked trains of five EAPs while performing simultaneous somatodendritic recordings. We restricted our analysis to physiologically relevant tonic firing frequencies (10/30/50 Hz) in these neurons. In TC neurons, our previous work using two-photon imaging indicated that neither frequency-dependent reduction in bAP-associated Ca^2+^ signals nor frequency-dependent initiation of dendritic Ca^2+^ spikes was a feature of these neurons (Errington et al., 2010). Our current work confirms these earlier findings, demonstrating no significant reduction in bAP amplitude (*n* = 25) or evidence of regenerative Ca^2+^ spikes across all frequencies tested (Fig. 6*A*,*B*). By dividing the amplitude of the fifth spike in the evoked train by the amplitude of the first spike for both the somatic and dendritic recordings, we found that frequency-dependent attenuation of spike amplitudes during trains was not observed at any distance from the soma for all spike frequencies in TC neurons (Fig. 6*C*). In TRN neurons, these properties have not been previously tested either directly by patch clamp or by imaging approaches. Similar to TC neurons, we observed no difference in bAP amplitude in TRN neurons (*n* = 7) throughout spike trains at each tested frequency (Fig. 6*D*,*E*). Once more, like TC neurons, frequency-dependent attenuation did not occur at any dendritic recording location at any frequency tested (Fig. 6*F*). Moreover, no evidence of bAP evoked dendritic Ca^2+^ electrogenesis, such as an increased spike afterdepolarization, was observed at frequencies up to 50 Hz (Fig. 6*D*).

In sum, these data demonstrate directly that, during tonic firing, APs backpropagate into the dendritic tree of both TC and TRN neurons but undergo strong voltage attenuation. In TC, but not TRN, neurons, membrane potential before spike onset can influence AP shape and duration and this produces variability in backpropagation efficiency. Moreover, the strength of AP backpropagation is not affected by either the frequency of firing or duration of the train, APs are always generated in the axosomatic region and TC and TRN dendrites do not support Na^+^-spike electrogenesis, most likely as a consequence of insufficient distal dendritic Na^+^ conductance.

### Distinct TC and TRN LTS burst properties determine AP backpropagation

**Figure 7.**
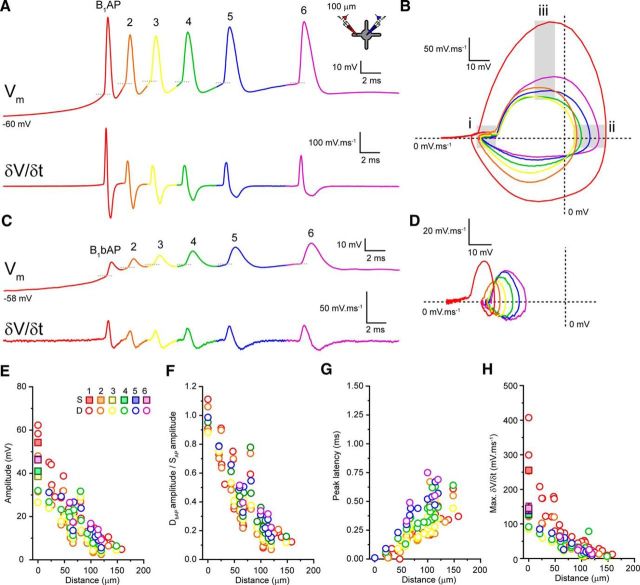
Backpropagation of APs during LTS bursts in TC neurons. ***A***, Somatic *V*_m_ and δ*V/*δ*t* of a TC neuron LTS burst. Individual APs within the burst (B_1_AP-B_6_AP) are color coded red to purple. ***B***, AP phase plot for the somatic LTS-burst APs shown in ***A***. Colors match the code in ***A***. Gray boxes highlight spike threshold (***i***), maximum amplitude (***ii***), and maximum δ*V/*δ*t* (***iii***). ***C***, Dendritic (B_1_bAP-B_6_bAP) *V*_m_ and δ*V/*δ*t* of the LTS burst shown in ***A***. ***D***, AP phase plot for the dendritic LTS burst shown in ***C***. Colors match the code in ***A***. ***E***, Amplitude of somatic and dendritic LTS-burst APs versus recording distance from soma. Somatic values are mean ± SEM. Inset shows the color coding for each spike in the train. ***F***, Normalized dendritic LTS-burst AP amplitude versus recording distance from soma. ***G***, Peak soma to dendrite latency versus distance from the soma for LTS-burst APs. ***H***, Maximum δ*V/*δ*t* versus distance from the soma for LTS-burst APs.

**Figure 8.**
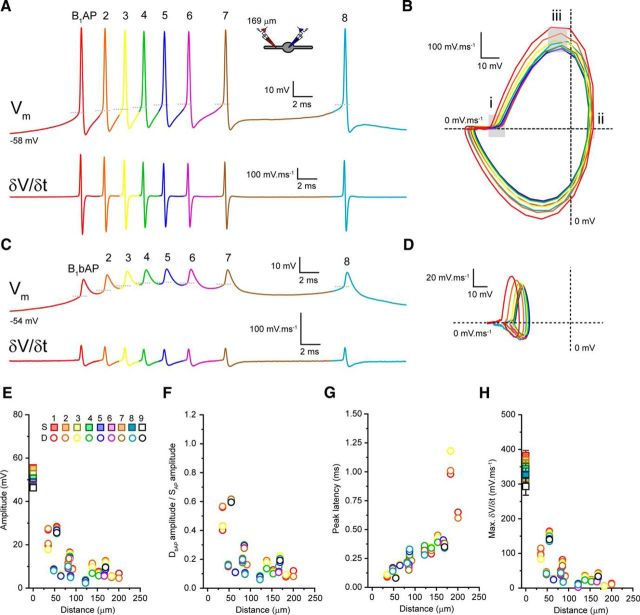
Backpropagation of APs during LTS bursts in TRN neurons. ***A***, Somatic *V*_m_ and δ*V/*δ*t* of a TRN neuron LTS burst. Individual APs within the burst (B_1_AP-B_8_AP) are color coded red to light blue. ***B***, AP phase plot for the somatic LTS-burst APs shown in ***A***. Colors match the code in ***A***. Gray boxes highlight: spike threshold (***i***), maximum amplitude (***ii***), and maximum δ*V/*δ*t* (***iii***). ***C***, Dendritic (B_1_bAP-B_8_bAP) *V*_m_ and δ*V/*δ*t* of the LTS burst shown in ***A***. ***D***, AP phase plot for the dendritic LTS burst shown in ***C***. Colors match the code in ***A***. ***E***, Amplitude of somatic and dendritic LTS-burst APs versus recording distance from soma. Somatic values are mean ± SEM. Inset shows the color coding for each spike in the train. ***F***, Normalized dendritic LTS-burst AP amplitude versus recording distance from soma. ***G***, Peak soma to dendrite latency versus distance from the soma for LTS-burst APs. ***H***, Maximum δ*V/*δ*t* versus distance from the soma for LTS-burst APs.

**Figure 9.**
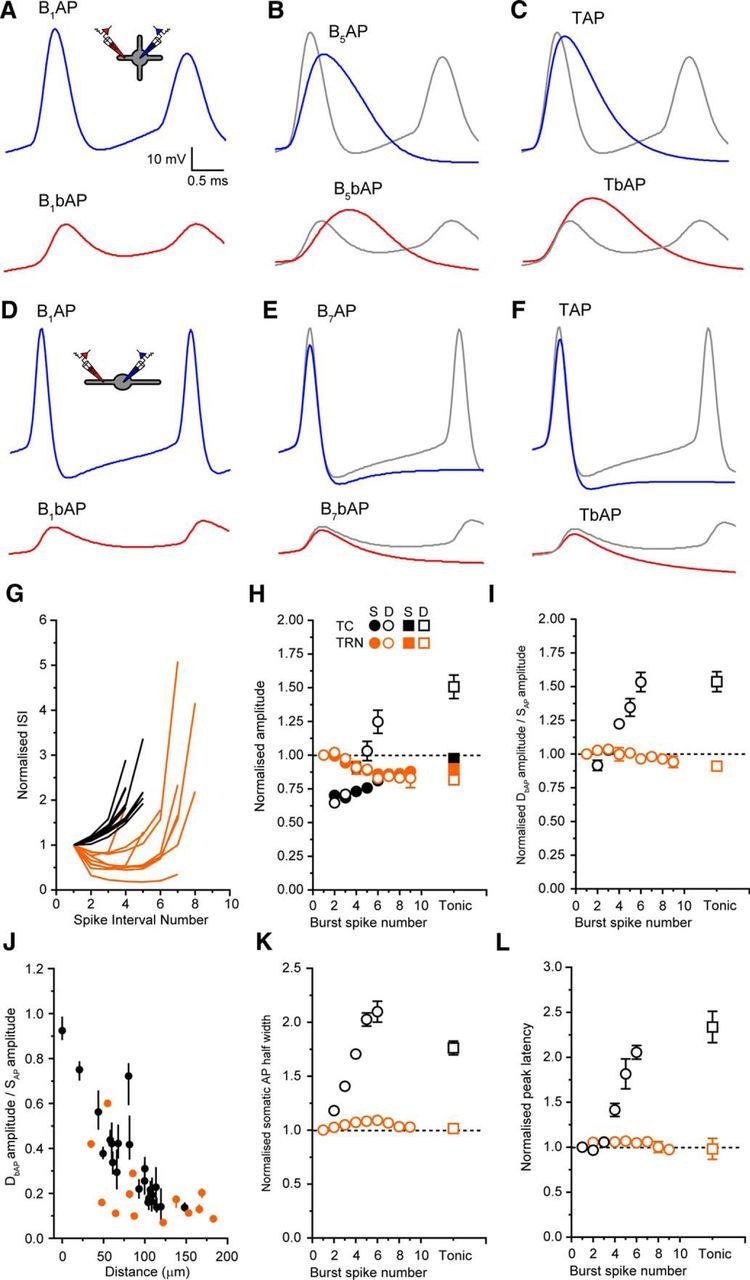
Variability of somatic spike shape influences backpropagation during bursts in TC but not TRN neurons. ***A***, Example traces showing the first spike of a typical LTS burst recorded in the soma (B_1_AP, blue) and dendrites (B_1_bAP, red) of a TC neuron. ***B***, B_5_AP (blue) and B_5_bAP (red) from the same LTS burst in ***A*** overlaid onto B_1_AP and B_1_bAP (gray). ***C***, Average TAP (blue) and TbAP (red) from the same neuron as in ***A*** overlaid onto B_1_AP and B_1_bAP (gray). ***D***, Example traces showing the first spike of a typical LTS burst recorded in the soma (B_1_AP, blue) and dendrites (B_1_bAP, red) of a TRN neuron. ***E***, B_7_AP (blue) and B_7_bAP (red) from the same LTS burst in ***D*** overlaid onto B_1_AP and B_1_bAP (gray). ***F***, Average TAP and TbAP from the same neuron in ***D*** overlaid onto B_1_AP and B_1_bAP (gray). ***G***, Interspike interval normalized to the interval between B_1_AP and B_2_AP for all LTS bursts of three or more spikes plotted against spike interval number for TC (black) and TRN (orange) neurons. ***H***, Somatic (filled symbols) and dendritic (open symbols) AP amplitudes for all LTS burst spikes normalized to the first spike in the burst for TC (black) and TRN (orange) neurons. ***I***, Dendritic spike attenuation (B_X_bAP/B_X_AP) normalized to the first spike in the burst for TC (black) and TRN (orange) neurons. ***J***, Dendritic spike attenuation versus distance from the soma for TC and TRN neurons showing the mean AP attenuation (circles) for all LTS burst spikes and the range of attenuation between APs within bursts (bars). ***K***, Somatic AP half-width for all LTS-burst APs normalized to the half-width of the first spike in the burst. ***L***, Peak latency for all LTS-burst APs normalized to the latency between B_1_AP and B_1_bAP.

So far, we have focused on tonic APs fired when thalamic neurons are depolarized. Although previous Ca^2+^-imaging studies (Crandall et al., 2010; Errington et al., 2010) suggest that LTS-burst APs contribute little to dendritic Ca^2+^ signaling in TC and TRN neurons, distal LTS-burst AP voltage responses remain to be described. Therefore, we investigated LTS-burst AP backpropagation in both types of thalamic neurons. To do this, we injected hyperpolarizing current into the soma of TC and TRN neurons and measured somatic (B_X_AP) and dendritic (B_X_bAP) rebound LTS-burst APs. Whereas the numbers of APs per burst varied in both TC (range: 1–6 spikes, 3.6 ± 0.3 spikes, *n* = 36) and TRN (range: 2–9 spikes, 5.5 ± 0.7 spikes, *n* = 16) neurons, all LTS bursts (3 or more spikes per burst) displayed the decelerating and accelerating–decelerating temporal firing patterns characteristic of TC and TRN neurons (Figs. 7*A*, 8*A*, 9*G*). When recording somatic LTS-burst APs, we found differences in individual spike properties throughout the burst that clearly distinguish TC from TRN neuron bursts. First, in TC neurons, the first spike, B_1_AP, has a substantially more hyperpolarized threshold than the next spike, B_2_AP, but the AP threshold varies little between B_2_AP and subsequent spikes in the burst (Fig. 7*A*). This threshold transition can be readily observed in the somatic AP phase plot (Fig. 7*B*). In comparison, as has been described *in vivo* (Munoz and Fuentealba, 2012), in TRN neurons, a much smaller, incremental increase in spike threshold occurs throughout the duration of the burst (Fig. 8*A*,*B*). Second, in TC neurons, as has been described previously (Turner et al., 1997, Williams and Stuart 2000), we saw a significant decrease in AP amplitude between B_1_AP (54.3 ± 1.1 mV, *n* = 36) and B_2_AP (39.8 ± 1.0 mV, *n* = 30, *p* < 0.0001, paired *t* test), followed by a progressive increase in amplitude for subsequent spikes (B_3_AP: 38.6 ± 1.2 mV, *n* = 23, B_4_AP: 41.0 ± 1.0 mV, *n* = 22; B_5_AP: 44.5 ± 1.4 mV, *n* = 12, B_6_AP: 46.3 ± 1.8 mV, *n* = 5; Fig. 7*A*), although late spikes never matched the amplitude of B_1_AP. Again, this is clearly illustrated by the AP phase plot (Fig. 7*B*). This is not a feature of APs during LTS bursts in TRN neurons, the amplitudes of which do not show significant variability throughout the burst (B_1_AP: 55.5 ± 1.3 mV, *n* = 16, B_2_AP: 55.0 ± 1.4 mV, *n* = 16, B_3_AP: 52.7 ± 1.6 mV, *n* = 13, B_4_AP: 50.7 ± 1.2 mV, *n* = 11, B_5_AP: 48.9 ± 1.1 mV, *n* = 9, B_6_AP: 47.7 ± 1.2 mV, *n* = 8, B_7_AP: 47.2 ± 1.4 mV, *n* = 7, B_8_AP: 47.0 ± 2.1 mV, *n* = 6, B_9_AP: 46.3 ± 3.4 mV, *n* = 2; Fig. 8*A*,*B*,*E*). This difference between TC and TRN neurons in somatic spike amplitude during bursts is shown clearly in Figure 9*H*, where somatic AP amplitude, normalized to B_1_AP, is shown for all bursts comprising three or more spikes. Third, and most critically, in TC neurons, we observed a progressive increase somatic spike half-width (B_1_AP: 0.42 ± 0.01 ms, *n* = 36, B_2_AP: 0.50 ± 0.01 ms, *n* = 30, B_3_AP: 0.58 ± 0.01 ms, *n* = 23, B_4_AP: 0.71 ± 0.02 ms, *n* = 22, B_5_AP: 0.84 ± 0.03 ms, *n* = 12, B_6_AP: 0.92 ± 0.06, *n* = 5; Fig. 7*A*,*B*) accompanied by a reduction in maximum AP δ*V/*δ*t* (Fig. 7*A*,*B*), highlighting a gradual slowing and broadening of spikes during the LTS burst. Normalizing the half-width of somatic LTS burst spikes to B_1_AP reveals a greater than twofold increase for bursts of five or more spikes (Fig. 9*K*). In fact, in TC neurons, comparing δ*V/*δ*t* of B_1_AP with subsequent spikes (Fig. 7*A*) reveals that early burst APs have shapes and durations more like a typical fast-spiking cell (cf. Figs. 7*A*, 8*A*, B_1_AP) and that, during bursts, a transformation of spike shape and duration occurs so that late burst spikes have properties similar to other regular spiking cells and to TC cells during tonic firing (Fig. 7*A*, B_1_AP–B_6_AP; Bean, 2007). In this respect, APs at the end of LTS bursts more closely resembled TAPs in terms of their amplitude (Fig. 9*A–C*,*H*) and half-width (Fig. 9*A–C*,*K*) than they did B_1_AP. In comparison, in TRN neurons, spike half-width (B_1_AP: 0.23 ± 0.01 ms, *n* = 16, B_2_AP: 0.24 ± 0.1 ms, *n* = 16, B_3_AP: 0.24 ± 0.01 ms, *n* = 13, B_4_AP: 0.23 ± 0.01 ms, *n* = 11, B_5_AP: 0.23 ± 0.01 ms, *n* = 9, B_6_AP: 0.23 ± 0.01 mV, *n* = 8, B_7_AP: 0.23 ± 0.01 ms, *n* = 7, B_8_AP: 0.22 ± 0.01 ms *n* = 6, B_9_AP: 0.24 ± 0.01 ms, *n* = 2; Fig. 8*A*,*B*) and maximum AP δ*V/*δ*t* (Fig. 8*A*,*B*) remained unaltered throughout the LTS burst, as demonstrated clearly by the somatic AP phase plot (Fig. 8*B*). Comparison of B_7_AP (Fig. 9*E*) of a TRN LTS burst with B_1_AP (Fig. 9*D*) illustrates the lack of change in spike amplitude (Fig. 9*H*) and half-width (Fig. 9*K*) during the burst. Moreover, APs during LTS bursts in TRN neurons were not different from TAPs in any of the measured parameters (Fig. 9*F*,*H*,*K*,*L*). Therefore, unlike TC neurons, TRN neuron APs are invariable and display fast-spiking properties in both LTS burst and tonic mode.

In TC neurons, the increase in somatic half-widths of LTS-burst APs were coincident with an increase in the efficiency of propagation into the dendrites. Similarly to their EAP and TAP counterparts, all LTS-burst APs in TC neurons from B_1_AP to B_6_AP showed a significant distance-dependent reduction in amplitude (Fig. 7*A–F*) and maximum δ*V/*δ*t* (Fig. 7*A–D*,*H*) as they propagated into dendrites. However, in contrast to somatic APs, in which the amplitude of spikes up to B_6_AP were reduced compared with B_1_AP (Fig. 7*A*), dendritic AP amplitudes were initially reduced, but subsequently increased such that, on average, B_5_bAP and B_6_bAP were in fact larger than B_1_bAP (Fig. 7*C*). Figure 9*H* illustrates clearly how B_5_bAP has a greater amplitude than B_1_bAP despite the fact that B_5_AP is smaller than B_1_AP. When considering these differences in somatic and dendritic AP amplitude during the LTS burst together, it is apparent that APs later in the LTS burst undergo weaker attenuation as they propagate into the dendrites (Fig. 7*F*). Comparing AP attenuation for subsequent LTS burst spikes against the attenuation experienced by B_1_bAP illustrates the increased backpropagation efficiency of late-burst spikes compared with their earlier counterparts (Fig. 9*A*,*B*,*I*). Indeed, λ_eff_ of B_1_bAP was 72 μm, whereas for B_5_bAP, it was 156 μm. Furthermore, the decrease in AP attenuation during the LTS burst was accompanied by an increase in the peak latency between somatic and dendritic APs (Fig. 7*A*,*C*,*G*). When the latency for each spike was normalized to the latency of the first LTS burst spike, we observed a greater than twofold increase in the latency between B_6_AP and B_6_bAP versus B_1_AP and B_1_bAP and clear similarity in the latency between late-burst spikes and TbAPs (Fig. 9*L*). Therefore, during LTS bursts in TC neurons, AP backpropagation strength is variable. B_1_AP, despite having a greater somatic amplitude than all subsequent APs in the burst, undergoes the strongest attenuation such that B_1_bAP is actually smaller than bAPs later in the burst despite their somatic amplitudes being less. The degree of attenuation is coincident with a marked broadening of the somatic APs during the burst.

In comparison, TRN neurons did not show variability in spike backpropagation during LTS bursts. Like EAPs and TAPs, all LTS burst spikes from B_1_AP through to B_9_AP showed significant distance-dependent attenuation as they propagated into the dendrites (Fig. 8*C*,*E*,*F*). As can be seen in Figures 8, *A* and *C*, and 9*H*, somatic and dendritic LTS-burst APs showed only a small reduction in amplitude compared with B_1_AP and B_1_bAP respectively as the burst progressed. Therefore, the distance-dependent AP amplitude attenuation across successive spikes remained virtually unaltered compared with the attenuation of B_1_AP and was nearly identical to the attenuation observed for TbAPs in TRN dendrites (Fig. 9*I*). In fact, λ_eff_ of B_1_bAP was 36 μm and, by B_7_bAP, it was unchanged at 39 μm. This is clearly illustrated by comparing B_1_bAP of a typical TRN burst with B_7_bAP of the same burst and the averaged TbAP from the same neuron (Fig. 9*D–F*). Furthermore, the lack of variability in attenuation strength between LTS-burst APs in TRN neuron dendrites was not related to the distance from the soma. Plotting both the mean and range of attenuation for LTS burst spikes against distance of the dendritic recording from the soma reveals that variability in AP attenuation during bursts is observed throughout the dendritic tree in TC neurons (black circles and bars), but not TRN neurons (Fig. 9*J*, orange circles and bars). Finally, in TRN neurons, the peak latency between somatic and dendritic APs, unlike TC neurons, was not altered for any LTS burst spikes or TbAPs when normalized to B_1_bAP (Fig. 9*L*).

## Discussion

The important outcomes of this study are as follows: (1) tonic and LTS-burst APs are generated in the axosomatic region in both TC and TRN neurons; (2) APs, in both firing modes in both TC and TRN neurons, backpropagate into the dendritic tree undergoing substantial distance-dependent voltage attenuation; (3) differences in somatic spike half-width between APs within LTS bursts and between LTS bursts and TbAPs result in variable AP backpropagation efficiency in TC, but not TRN, neurons; and (4) neither TC or TRN neuron dendrites support generation of local dendritic Na^+^ spikes. When considered alongside our own previous studies (Errington et al., 2010, 2012; Connelly et al., 2015, 2016) and those of others (Crandall et al., 2010, Cueni et al., 2008, Chausson et al., 2013), these new findings allow us to build a more complete picture of the role that dendrites play in thalamic neuron electrogenesis and signaling.

First, using somatodendritic recording, we established that both tonic and LTS-burst APs are recorded first at the soma and subsequently in the dendrites in both TC and TRN neurons. This demonstrates that, consistent with most other mammalian neurons (Clark et al., 2005; Kole et al., 2008; Schmidt-Hieber et al., 2008; Foust et al., 2010), APs are initiated in the perisomatic region, most likely the axon initial segment. For TC neurons, these results support those previously reported by Williams and Stuart (2000), but this is the first direct demonstration of axosomatic AP origin in TRN neurons. Ultrastructural studies have shown previously that axons occasionally originate from proximal dendrites rather than the soma in TRN neurons (Pinault and Deschênes, 1998). Although our limited number of dendritic recordings cannot exclude the possibility that spike initiation may occur in axons branching from dendrites rather than the soma, the fact that we observed no cases where APs were recorded first in the dendrite indicates that APs are triggered close to the soma and not from some distal part of the dendritic tree. From a physiological and computational perspective, it appears to be of little significance whether APs are generated in an axon that originates at the soma or from a proximal dendrite. Moreover, comparing the peak latency between somatic and dendritic APs confirms that, unsurprisingly, APs fired on the crest of low-threshold Ca^2+^ spikes originate from the same subcellular location as tonic APs.

By recording directly from dendrites, our new findings establish definitively the distance dependence of AP backpropagation in TC and TRN. In TC neurons, dendritic membrane voltage recordings strongly support previous findings from two-photon Ca^2+^ imaging studies (Crandall et al., 2010; Errington et al., 2010, 2012; Sieber et al., 2013). We found that tonic APs undergo strong voltage attenuation as they backpropagate into the dendritic tree, having amplitudes only 10–20% of those recorded at the soma once they reach the dendritic tips. This is consistent with larger bAP-evoked Ca^2+^ signals in proximal parts of the dendritic tree and suggests that the absence of bAP-evoked Ca^2+^ signals in distal branches (Crandall et al., 2010; Errington et al., 2010, 2012; Sieber et al., 2013) results from weak Na^+^ conductance-dependent active backpropagation coupled with strong passive dendritic filtering rather than a lack of distal dendritic high-voltage-activated Ca^2+^ channels. Moreover, because bAPs do not undergo any frequency-dependent reduction in their amplitude, they can reliably signal cellular spike output to the dendritic tree even for high firing frequencies. In TRN neurons, we also observed strong voltage attenuation of APs as they backpropagated into the dendritic tree. In this respect, our data from the first direct patch-clamp recordings of APs in TRN neuron dendrites more closely support the conclusion that bAP-evoked Ca^2+^ signals are minimal in distal dendrites (Cueni et al., 2008; Crandall et al., 2010) than those of Chausson et al. (2013), who reported bAP-evoked Ca^2+^ entry throughout much of the dendritic tree. The reasons for these discrepancies remain unclear, but might relate to differences in experimental conditions. For example, the duration of the AP trains in the study by Chausson et al. (2013) were typically much longer, introducing the possibility that the distal dendritic Ca^2+^ signals that they observed were due to slow axial dendritic diffusion rather than local Ca^2+^ entry.

APs are efficient triggers for neurotransmitter release at both axonal and dendritic presynaptic terminals (Bischofberger and Jonas, 1997). In TRN, GABAergic synapses between dendrites have been hypothesized to exist based on electron microscopy studies, although their functional existence remains to be demonstrated. We demonstrate here that APs attenuate significantly as they propagate into TRN neuron dendrites, producing depolarizations of only 10 mV or less at distances >100 μm from the soma. Therefore, if AP-triggered neurotransmitter release occurs at dendrodendritic synapses between TRN neurons, then it is probable that those synapses will be located close to the soma. Conversely, dendrodendritic synapses may be located at more distal locations, but in this case, given their robust attenuation, it is unlikely that bAPs would trigger release at these synapses. In contrast, release at distal dendrodendritic synapses may be triggered by low-threshold Ca^2+^ spikes, which as we have shown previously, are associated with global dendritic depolarization (Connelly et al., 2015). Importantly, therefore, signaling at dendrodendritic synapses in TRN, if it occurs, may show marked behavioral state dependence.

Functional evidence for the existence of dendrodendritic chemical synapses in TRN remains absent. In comparison, the functional evidence for the presence of electrical synapses formed by Cx36-dependent gap junctions between TRN dendrites is far more convincing (Landisman et al., 2002; Landisman and Connors, 2005; Lee et al., 2014). These electrical synapses transmit both slow membrane potential changes and fast APs between TRN neurons. Based on paired somatic recordings, the efficiency of transmission of fast Na^+^ spikes through TRN gap junctions is reasonably low. However, dye-coupling experiments also demonstrate the presence of coupled cells with intrasomatic distances of several hundred micrometers indicating coupling between distal dendrites (Lee et al., 2014). Therefore, although gap junctions themselves act as low-pass filters, the attenuation of spike amplitude by TRN dendrites is also likely to have a critical role in electrical synaptic transmission. Considering the strong soma to dendrite attenuation of fast signals such as APs in TRN dendrites alongside the highly efficient soma to dendrite transfer of slow voltage signals and the global mechanism of LTS generation (Connelly et al., 2015), it seems likely that these dendrites are optimized for slower electrical signaling. Conversely, because high local dendritic input impedance (Connelly et al., 2015) produces large local EPSPs, these synaptic potentials may be transmitted between TRN neurons through distal gap junctions. Whereas, as a result of strong dendrite to soma voltage attenuation (Connelly et al., 2015), these potentials may not individually be sufficient to influence somatic membrane potential, they could be integrated with other local chemical and electrical synaptic signals to control cellular output.

We have shown here in TC neurons that somatic AP half-width is influenced by the preceding membrane potential with important consequences for AP backpropagation. When APs were evoked from a resting membrane potential of −55 mV, their half-width was significantly shorter than when fired from a more depolarized potential close to spike threshold. This pattern is also observed during an LTS burst, in which the first spike in the burst is significantly faster than the fifth or sixth spike. The physiological significance of this difference in AP backpropagation between burst and tonic firing in TC, but not TRN, neurons remains to be determined. It is possible that these differences in spike shape are more significant to the axonal output of thalamic neurons rather than having a major impact on dendritic signaling. For example, at the mossy fiber-CA3 synapse in the hippocampus, repetitive axonal stimulation leads to broadening of APs in the presynaptic terminal and enhanced neurotransmitter release via inactivation of A-type K^+^ channels (Geiger and Jonas, 2000). Conversely, during TC neuron bursts, the initial spike in the burst is thought to have the greatest efficacy in driving postsynaptic neocortical spikes, whereas subsequent spikes in a burst are no more efficacious than their tonic counterparts (Swadlow and Gusev, 2001).

In this study, we have demonstrated directly for the first time that APs undergo strong voltage attenuation as they propagate into the dendrites of both excitatory glutamatergic TC and inhibitory GABAergic TRN neurons, but that state-dependent variability exists between tonic and LTS-burst dendritic AP signaling in TC neurons that is absent TRN neurons.
